# Low-grade appendiceal mucinous neoplasm associated with Urothelial carcinoma: A rare case report from Syria

**DOI:** 10.1016/j.amsu.2022.103525

**Published:** 2022-03-29

**Authors:** Moatasem Hussein Al-janabi, Tala Zidan, Eman Zidan, Muhammad Sinan Muhammed, Rabab Salloum

**Affiliations:** a Department of Pathology, Cancer Research Center, Tishreen University Hospital, Lattakia, Syria; b Tishreen University Faculty of Medicine, Lattakia, Syria; c Department of Urologic Surgery, Tishreen University Hospital, Lattakia, Syria; d Department of Pathology, Tishreen University Hospital, Lattakia, Syria

**Keywords:** Appendix, Mucinous neoplasm, Low-grade, LAMN

## Abstract

**Introduction:**

and importance: Low-grade appendiceal mucinous neoplasm is a rare neoplasm found in the appendix vermiform. Appendiceal mucinous neoplasms are usually seen by accident during investigations or surgeries for other reasons in the abdomen. Low-grade appendiceal mucinous tumors have no specific symptoms.

**Case presentation:**

We report an unusual case of a 72-year-old man with low-grade appendiceal mucinous neoplasm, which was found accidently during the investigation of the bladder tumor on a CT scan. Pathological examination of the specimen showed a cystic dilation at the distal tip of the appendix, filled with gelatinous mucus. Microscopically, we found mucinous epithelial proliferation with extracellular mucin.

**Clinical discussion:**

Low-grade appendiceal mucinous neoplasm is an uncommon tumor of the appendix. The incidence of low-grade appendiceal mucinous neoplasm is reported to be 1% of all tumors of the gastrointestinal tract and is found in less than 0.3% of appendectomy specimens. Appendiceal mucinous tumors are usually found with no specific symptoms.

**Conclusion:**

Low-grade appendiceal mucinous neoplasms are rare tumors of the gastrointestinal tract. The appropriate management for this tumor is appendectomy. The most feared complication of these tumors is seeding of mucin into the peritoneal cavity, leading to pseudomyxoma peritonei.

## Highlights

• Low-grade appendiceal mucinous neoplasm (LAMN) is a rare tumor found in the appendix vermiform.
• LAMNs may progress to pseudomyxoma peritonei, which is the most troubling complication.
• We report a case of a 72-year-old man with LAMN, found during the investigation of the bladder tumor on a CT scan.

## 1 Introduction

Low-grade appendiceal mucinous neoplasm (LAMN) is an infrequent tumor making appendix bulbous enlargement due to the secretion of abundant gelatinous mucin. This tumor is a rare entity found in 0.3% of appendiceal specimens [1,2]. The average age at diagnosis of the disease is 70 years and more common in women [3]. The symptoms of LAMN are often atypical and discovered accidently during investigations for other cases [4]. In this report, we present an unusual case of a 72-year-old man with low-grade appendiceal mucinous neoplasm, which was found during radical cystectomy.

This case report has been reported in line with the SCARE criteria 2020 [5].

## 2 Case presentation

**Fig. 1:**
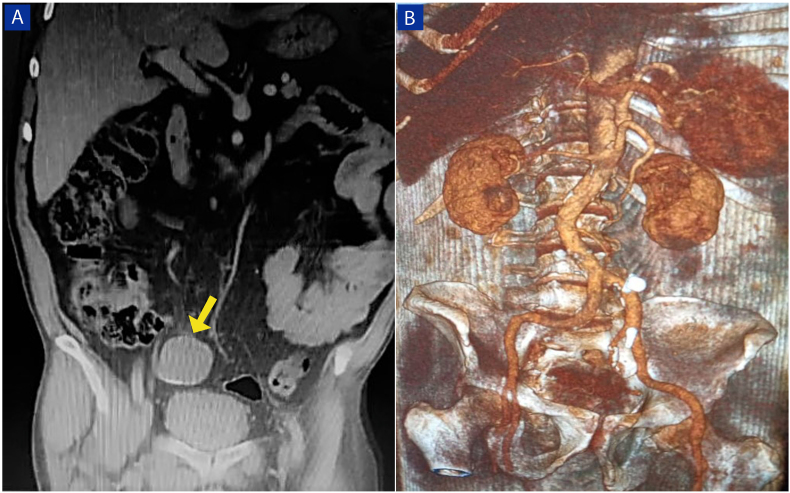
(A) Coronal contrast-enhanced CT image shows a heterogeneous mass in the right lower quadrant in the expected location of the appendix with tiny peripheral calcifications (yellow arrow), associated with bladder tumor. (B) A three-dimensional CT angiographic image demonstrates the integrity of the right common iliac artery. . (For interpretation of the references to colour in this figure legend, the reader is referred to the Web version of this article.)

**Fig. 2:**
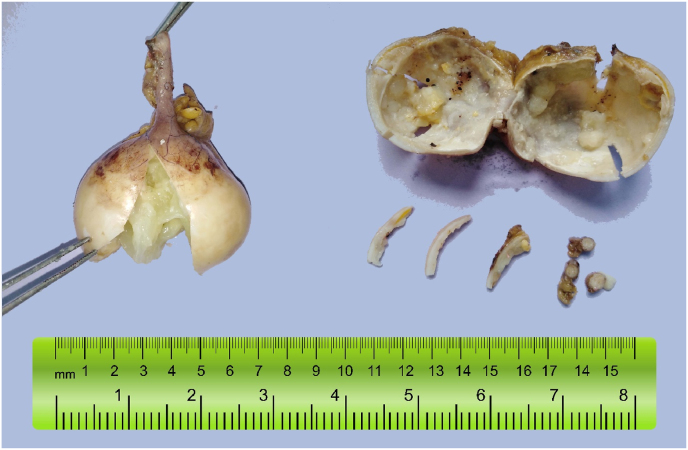
Gross image of excised appendix vermiform showing enlarged tumor at the tip of the appendix with mucus and tiny calcifications in the lumen. The base of the appendix has a normal appearance.

**Fig. 3:**
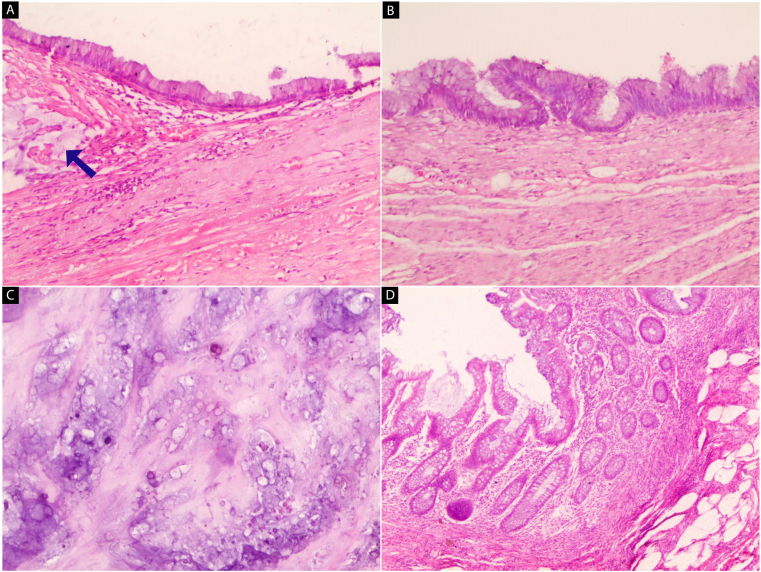
H&E stain (A–D): Microscopic images of the appendix vermiform with mucinous tumor at the tip. (A) Mucinous epithelial proliferation with extracellular mucin (blue arrow) (x 40). (B) The epithelium rests on mural fibrosis (x 100). (C) Foci of mucin and calcifications (x 200). (D) The Base of the appendix is within normal limits (x 100). . (For interpretation of the references to colour in this figure legend, the reader is referred to the Web version of this article.)

A 72-year-old man was referred to Tishreen University Hospital in 2021 to perform a radical cystectomy due to Urothelial carcinoma with muscularis propria invasion, which was confirmed by histopathological biopsy. Laboratory tests showed a low hemoglobin level; 10.2 g/dL with hematuria. Other Routine blood values were normal. The patient medical history shows that he underwent transurethral resection of bladder tumor (TURBT) followed by intravesical BCG (Bacillus Calmette-Guerin) therapy administered for 3 years. The patient has smoked 5 cigarettes daily for the past 40 years. He had never used alcohol. He had no allergy to any drugs. There was no tumor history in his family; particularly no history of bladder tumors. Computerized tomography (CT) of the abdomen and pelvis with contrast was performed. Coronal contrast-enhanced CT image shows a mass occupying almost the entire bladder cavity, in addition to the presence of a heterogeneous mass in the right lower quadrant in the expected location of the appendix with tiny peripheral calcifications (Fig. 1A). That mass was suspected of being a right common iliac artery aneurysm, but the CT angiography (CTA) showed the integrity of the artery (Fig. 1B). The patient underwent a radical cystectomy and appendectomy. Both specimens were sent to the pathology department for histopathological study. Pathological examination of the bladder specimen showed the tumor was a high-grade invasive carcinoma that invaded all layers of the bladder and reached perivesical tissue. On the other hand, the gross examination of the appendix specimen revealed a cystic dilation at the distal tip, measured 5.5 cm × 4.5 cm x 5 cm, filled with gelatinous mucus, and some calcifications were seen in the lumen. The serosa was smooth. (Fig. 2). Microscopically, we found mucinous epithelial proliferation with extracellular mucin (Fig. 3A), and the epithelium rests on mural fibrosis (Fig. 3B). Foci of mucin and calcifications are seen (Fig. 3C). While the base of the appendix is within normal limits (Fig. 3D). The patient was stable and discharged 15 days later, without any complications.

## 3 Discussion

Low-grade appendiceal mucinous neoplasm (LAMN) is an uncommon tumor of the appendix due to much secretion of mucus and accumulation in the lumen which leads to the cystically vast appendix. The incidence of low-grade appendiceal mucinous neoplasm is reported to be 1% of all tumors of the gastrointestinal tract and is found in less than 0.3% of appendectomy specimens [4]. Appendiceal mucinous tumors are usually found with no specific symptoms. However, some patients present with right lower quadrant pain or acute appendicitis. In addition, these tumors may misdiagnose as retroperitoneal or right adnexa masses, especially in females [4,6]. In most cases, these neoplasms are discovered by accident during investigations for other reasons in the abdomen [6,7], like our case. As for the diagnostic methods that help in detecting the tumor, ultrasonography and CT can clearly show the size of the appendiceal tumor, its appearance, the amount of mucus in the lumen, and its anatomical relationships [8]. However, a CT scan is more sensitive than echography in evaluating the tumor and detecting the parietal calcifications, which is highly suggestive of the diagnosis, but it is revealed only in 50% of cases [8,9]. Surgical resection of low-grade appendiceal mucinous neoplasms is recommended [10]. The most important point in the management of LAMNs is to avoid their rupture and mucin fills the abdomen which leads to pseudomyxoma peritonei, a rare malignant tumor, characterized by the accumulation of abundant mucin in the peritoneum cavity [4,10]. Despite low-grade appendiceal mucinous neoplasm being a slow-growing neoplasm, both LAMN and mucinous adenocarcinoma may progress to the pseudomyxoma peritonei [3,4].

## 4 Conclusion

Low-grade appendiceal mucinous neoplasms (LAMNs) are rare tumors of the gastrointestinal tract. The treatment of choice for LAMN is surgical excision. Although LAMN is slow-growing, it has the risk of rupturing either spontaneously or due to surgery, leading to seeding of mucin into the peritoneum, forming pseudomyxoma peritonei, which is the most troubling complication.

## Provenance and peer review

Not commissioned, externally peer-reviewed.

## Sources of funding

This research did not receive any specific grant from funding agencies in the public, commercial, or not-for-profit sectors.

## Ethical approval

No ethical approval was needed for this case report.

## Registration of research studies

Not applicable.

## Guarantor

Rabab Salloum.

## Consent for publication

Written informed consent was obtained from the patient for publication of this case report and accompanying images. A copy of the written consent is available for review by the Editor-in-Chief of this journal.

## Author contribution

Moatasem Hussein Al-janabi: participated in study design, data collections, data analysis, and writing. Tala Zidan: participated in study design, data analysis, and writing. Eman Zidan: participated in study design, data analysis, and writing. Muhammad Sinan Muhammed: performed this surgery and data collection. Rabab Salloum: in reviewing the manuscript.

## Declaration of competing interest

The authors have no conflicts of interest to declare.
